# Unlocking phosphorus resources: phosphate-solubilizing microorganisms as a green strategy for activating phosphorus in acidic red soils and promoting crop growth

**DOI:** 10.3389/fmicb.2025.1630650

**Published:** 2025-07-09

**Authors:** Qingqing Ma, Huayi Chen, Yuting Yang, Bin Zhou

**Affiliations:** ^1^Tropical Crops Genetic Resources Institute, Chinese Academy of Tropical Agricultural Sciences, Haikou, Hainan, China; ^2^School of Tropical Agriculture and Forestry, Hainan University, Haikou, China

**Keywords:** microbial technology, red soil improvement, phosphorus inactivation, agricultural sustainability, tropical and subtropical agriculture

## 1 Introduction

Tropical and subtropical agriculture is crucial for global food and economic crop production, and soil health directly impacts regional ecological security and food output. Red soils, widely distributed in these regions, are typical soil types formed by the long-term weathering of iron- and aluminum-rich oxides in hot and humid climates. Their red or brownish-red color results from the accumulation of iron and aluminum oxides. For instance, the red soil region in South China, covering 2.18 × 10^6^ km^2^ (about 36% of China's arable land), is key for economic and food crop production (Lang et al., 2025; Huang and Zhao, 2014). However, due to their strong acidity, low base saturation, and high phosphorus (P) fixation capacity, red soils are among the most vulnerable soil types in agricultural production (Long et al., 2024).

P is essential for plant growth and development, driving cell division, differentiation, and metabolic processes (Ashley et al., 2011). While weathering of red soil parent material releases a significant amount of P, its bioavailability is constrained by the unique formation process of soil and mineral characteristics, resulting in a low flux biogeochemical cycle of P in red soil ecosystems (Ma et al., 2016). Under acidic conditions, secondary clay minerals and amorphous iron and aluminum oxides expose numerous hydroxyl sites, which adsorb phosphate ions specifically, forming stable inner-sphere complexes. Consequently, P is fixed in soil solids as amorphous or crystalline iron- and aluminum-phosphate (He et al., 2025). This chemical fixation is largely irreversible, creating a surplus of fixed P and significantly limiting the flux of available P in the soil-plant system (Huang et al., 2021). Moreover, the low organic matter content in red soils weakens the P coordination-dissolution balance. Soil aggregate destruction caused by intensive farming further exacerbates P leaching into deep soil layers (Huang et al., 2021). These factors, which cause P deactivation, severely restrict agricultural production in red soils. Critically, sustainable alternatives remain underexplored, particularly regarding microbiome-driven P activation. This review uniquely bridges microbial ecology with agronomic application, highlighting how phosphate-solubilizing microorganisms (PSMs) concurrently resolve P limitation, aluminum toxicity, and soil structural decline-gaps unaddressed by chemical approaches.

## 2 PSMs have unique potential in red soil improvement

### 2.1 Microbial technologies are widely used in red soil improvement

In the global context of sustainable agriculture, microbial technology, known for being eco-friendly and functionally diverse, has become a key research focus in red soil improvement. Traditional chemical methods, such as lime application and chemical phosphate fertilizer addition, can temporarily adjust soil acidity or supplement P (Table 1). However, their long-term use may lead to problems like secondary salinization, soil structure degradation, and increased ecological risks (Ji et al., 2024). In contrast, microbial technology can regulate soil biogeochemical cycles, thereby achieving multiple goals of nutrient activation, toxicity mitigation, and ecological restoration. Microbial metabolism directly acts on soil minerals and organic matter, releasing fixed nutrients and enhancing the soil microenvironment via metabolic products (Khan et al., 2024). Additionally, microbial growth promotes soil aggregate formation, thereby improving soil structure and boosting water and nutrient retention capacities (Long et al., 2025).

**Table 1:** Methods of red soil improvement and their advantages and drawbacks.

**Types**	**Technologies**	**Advantages**	**Drawbacks**	**References**
Chemical	Liming	• Quickly neutralizes soil acidity • Reduces aluminum toxicity	• Excessive use can lead to soil compaction	Xu et al. (2023)
	Oyster shell Powder	• Neutralizes soil acidity • Supplies calcium and magnesium	• Slow improvement rate • Influenced by soil conditions	Li et al. (2025)
	Phosphogypsum	• Neutralizes acidity • Provides calcium and sulfur	• Contains heavy metals and harmful substances	Sun et al. (2025)
	Biochar	• Neutralizes acidity • Improves soil fertility	• Affects soil infiltration • Uncertain long-term effects	He et al. (2025)
Physical	Deep tillage	• Enhances soil aeration and permeability • Promotes microbial activity	• Mechanical compaction may cause local soil compaction	Chen et al. (2020)
Biological	Microbial inoculant	• Increases beneficial microorganisms • Promotes nutrient transformation	• Microbial growth is affected by environmental conditions	Shi et al. (2024)
	Plant-based remediation	• Lowers heavy metal toxicity • Improves soil ecosystem	• Long restoration period • Plant species selection is crucial	Li et al. (2024)
Agronomic Practices	Organic manure	• Raises organic matter content • Improves soil structure	• Slow nutrient release • Fails to meet immediate crop needs	Qiu et al. (2025)
	Crop rotation	• Improves soil physical and chemical properties • Reduces pests and diseases	• Rotation mode selection is complex	Mao et al. (2025)

### 2.2 PSMs as potential candidates for red soil amelioration

PSMs are beneficial functional microorganisms that convert insoluble environmental phosphorus into plant-available forms. They were first identified in farmland and environmental studies (Vassilev et al., 2006). PSMs secrete acids and extracellular enzymes to dissolve insoluble inorganic and organic phosphates. Additionally, they release intracellular P through cell lysis, thereby enhancing soil P availability and improving soil microbial community balance (Hu and Chen, 2023). These characteristics provide PSMs with a unique advantage in red soil improvement. Notably, their ecological adaptability enables them to remain active under the extremely acidic conditions of red soils. For instance, studies have shown that Pseudomonas duriflava, Enterobacter quasimori, and Acinetobacter sp. exhibit robust phosphorus-solubilizing abilities at pH 4.5–6.2, indicating their genetic potential to adapt to red soil acidification (Kumar et al., 2025; Hidayat et al., 2024; Liu et al., 2014). Importantly, PSMs can maintain soil P availability over time, providing sustained nutrient support for crop growth, reducing reliance on external phosphate fertilizers, and thus aligning with the goals of red soil improvement and sustainable agriculture (Rawat et al., 2021).

## 3 PSMs regulate community structure to enhance red soil fertility via P activation

**Figure 1:**
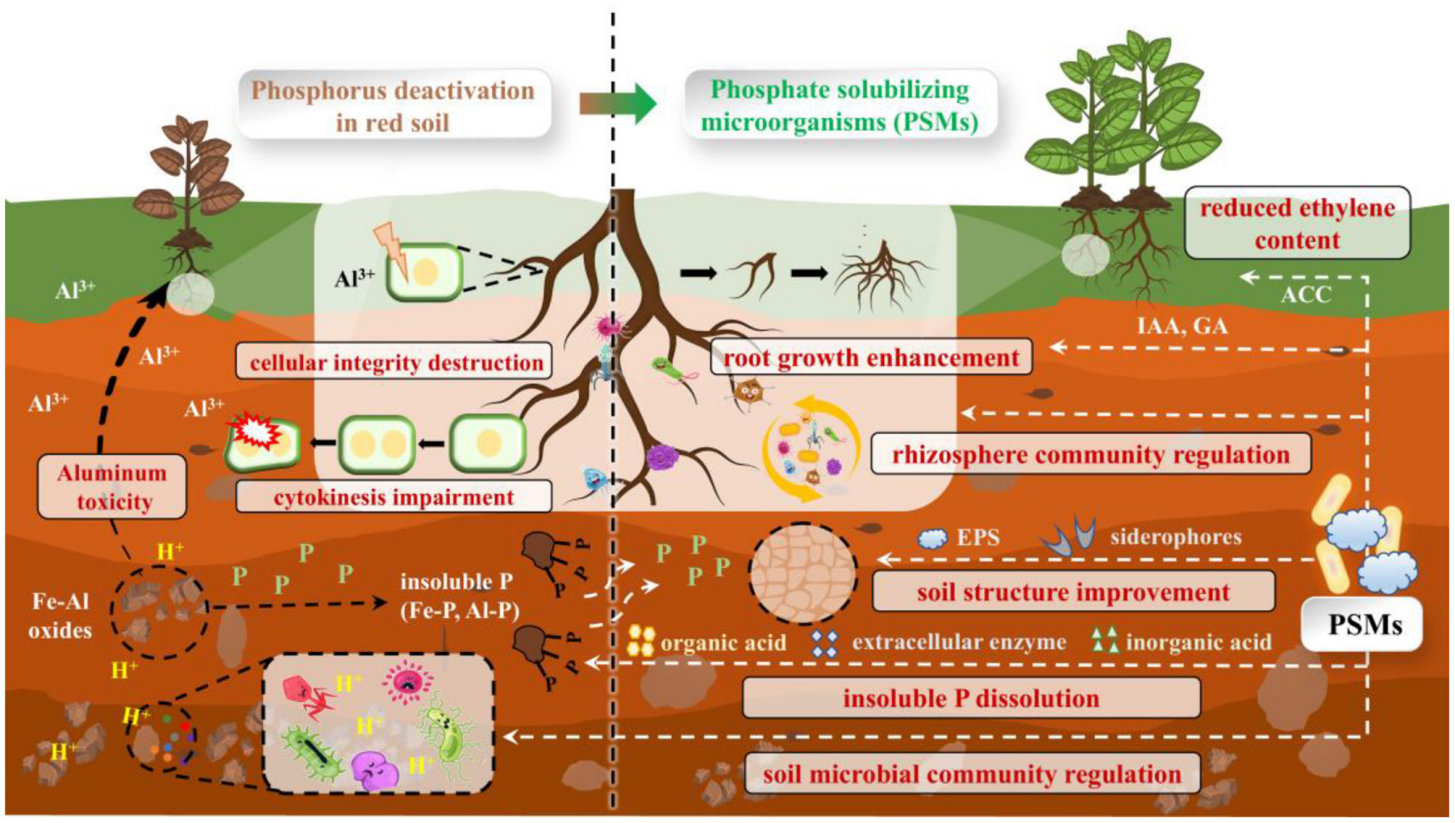
Schematic diagram of phosphate-solubilizing microorganisms improving red soil and crop growth.

PSMs improve red soil fertility not only through their inherent functions but also by regulating the soil microbial community to form a synergistic ecological network (Figure 1). The decline in microbial diversity and functional imbalance caused by red soil acidification are key factors limiting nutrient cycling. Introduced PSMs can reshape the rhizosphere microbiome while modifying the local microenvironment. For example, they promote the colonization of beneficial bacteria, such as nitrogen-fixing bacteria, and boost the relative abundance of function microbes related to P cycling (Rawat et al., 2021). This community optimization builds a metabolic module centered on P activation, thereby strengthening the transformation efficiency of soil nutrients. Specifically, organic acids, inorganic acids, and enzymes secreted by PSMs can solubilize occluded P by protonating Fe-P and Al-P surfaces, thereby increasing available soil P and alleviating aluminum toxicity (Ahmad et al., 2023). Moreover, PSM metabolic products (e.g., exopolysaccharides, siderophores) stabilize soil aggregates, thereby improving pore structure and water-holding capacity and creating a favorable habitat for microbial activity (Khan et al., 2024).

## 4 PSMs boost crop yield and stress resistance in red soils through multifaceted promotion

The effects of PSMs on red soil improvement and crop promotion are presented in Table 2. The growth-promoting effects of PSMs on red soil crops extend beyond mere nutrient supply. By multidimensionally regulating plant physiology, PSMs enhance crops' environmental adaptability, ensuring stable and increased yields under stress. Under red soil's strong acidity and aluminum toxicity, PSMs secrete plant hormones such as indoleacetic acid and gibberellins. These hormones stimulate root meristem activity, promoting lateral root and root hair development, thereby expanding nutrient absorption (Masrahi et al., 2023; Feng et al., 2024). Meanwhile, the ACC deaminase produced by PSMs degrades the ethylene precursor generated under plant stress, thereby alleviating root growth inhibition (Kumar et al., 2025). When confronting oxidative stress, PSMs activate the antioxidant enzyme system in crops (e.g., superoxide dismutase, peroxidase), scavenging excess reactive oxygen species and maintaining cell membrane stability (Rawat et al., 2021). Furthermore, the mutualistic relationship between PSMs and crop roots can induce systemic resistance in plants, thereby strengthening defense against soil-borne pathogens. For instance, combined amino acid and PSM applications have reduced pathogenic fungal relative abundance by 5.2%, thereby lowering disease risk (Shi et al., 2024). This mutualistic relationship involves complex signaling pathways and metabolic interactions. PSMs produce specific molecules that trigger plant defense responses, such as the production of pathogenesis-related proteins and secondary metabolites (Guo et al., 2024). In field practices, PSM applications consistently improve crop growth, foliar nitrogen and phosphorus concentrations, grain quality, and yields (Masrahi et al., 2023). This comprehensive regulation, ranging from soil improvement to enhancing crop stress resistance, highlights PSMs' central value in red soil agro-ecosystems.

**Table 2:** Phosphorus solubilizing capacity of PSMs and its effect on red soil improvement and crop growth.

**PSM strains**	**Genus**	**P solubility (mg/L)**	**Soil available P improvement**	**Crop types**	**Crop growth**	**References**
ASL12	*Acinetobacter* sp.	717	51.27%	*Areca catechu*	82.84% increase in plant height	Liu et al. (2014)
ASG33	*Shigella* sp.	530	/	/	/	
ASG34	*Escherichia* sp.	499	34.32%	*Areca catechu*	74.90% increase in plant height	
ADH302	*Enterobacter* sp.	426	24.15%	*Areca catechu*	71.55% increase in plant height	
ATZ304	*Paenibacillus* sp.	266	/	/	/	
ASG64	*Paenibacillus* sp.	165	/	/	/	
ADH306	*Bacillus* sp.	137	/	/	/	
ASG16	*Kurthia* sp.	439	/	/	/	
ASG41	*Paenibacillus* sp.	300	/	/	/	
AX7	*Rhizobium* sp.	275	/	/	/	
NBW	*Bacillus subtilis*	/	81.0%	Pepper	66.50% increase in yield	Duan et al. (2024)
PSB 5	/	44.4	/	/	/	Kumar et al. (2025)
PSB 8	/	48.0	/	/	/	
PSB 9	/	44.7	/	/	/	
PSB 10	/	53.4	/	/	/	
13-2	*Bacillus amyloliquefaciens*	/	/	Tomato	21.38% increase in plant height	Guo et al. (2024)
RW37	*Enterobacter soli*	498	/	Moso bamboo	56.49% increase in plant height	Zhang et al. (2024)

## 5 Future research directions and strategies

PSMs activate soil P reserves, alleviate aluminum toxicity, and secrete growth promoters, representing a cost-effective and sustainable green strategy for red soil improvement. However, given some limitations of PSMs, future research can be further deepened in the following aspects: Firstly, targeting the extreme acidity of red soil, screening and engineering functional strains with efficient P solubilization and acid tolerance is crucial. Decoding the acid-resistant molecular mechanisms of PSMs via genomics and metabolomics, combined with gene editing to enhance their phosphorus-solubilizing capacity or aluminum detoxification pathways, can boost strain adaptability. Secondly, developing integrated restoration systems that combine multiple technologies is essential to overcome the limitations of single microbial technologies. For example, combining PSMs with biochar, phosphate minerals, and harmless phosphogypsum can provide microbial habitats and synergistically regulate soil pH. Additionally, it is necessary to comprehensively evaluate the long-term impacts of PSM application on the structure and functions of soil microbial communities. This can prevent potential imbalances or functional redundancies in indigenous microbial populations that may be caused by the introduction of exogenous microorganisms.
